# ECCB2024: The 23rd European Conference on Computational Biology

**DOI:** 10.1093/bioinformatics/btae422

**Published:** 2024-09-04

**Authors:** Anu Kukkonen-Macchi, Sampsa Hautaniemi, Katharina F Heil, Merja Heinäniemi, Lars Juhl Jensen, Sini Junttila, Lukas Käll, Asta Laiho, Peter Maccallum, Matti Nykter, Bengt Persson, Tomi Suomi, Tim Van Den Bossche, Tommi H Nyrönen, Laura L Elo

**Affiliations:** Turku Bioscience Centre, University of Turku and Åbo Akademi University, 20520 Turku, Finland; Research Program in Systems Oncology, Faculty of Medicine, University of Helsinki, 00014 Helsinki, Finland; ELIXIR, Wellcome Genome Campus, Hinxton, Cambridge, CB10 1SD, United Kingdom; The Institute of Biomedicine, School of Medicine, University of Eastern Finland, 70210 Kuopio, Finland; Novo Nordisk Foundation Center for Protein Research, Faculty of Health and Medical Sciences, University of Copenhagen, 2200 Copenhagen N, Denmark; Turku Bioscience Centre, University of Turku and Åbo Akademi University, 20520 Turku, Finland; Science for Life Laboratory, KTH – Royal Institute of Technology, Stockholm 17165, Sweden; Turku Bioscience Centre, University of Turku and Åbo Akademi University, 20520 Turku, Finland; ELIXIR, Wellcome Genome Campus, Hinxton, Cambridge, CB10 1SD, United Kingdom; Prostate Cancer Research Center, Faculty of Medicine and Health Technology, Tampere University, 33520 Tampere, Finland; Department of Cell and Molecular Biology, Science for Life Laboratory, Uppsala University, 751 24 Uppsala, Sweden; Turku Bioscience Centre, University of Turku and Åbo Akademi University, 20520 Turku, Finland; VIB-UGent Center for Medical Biotechnology, VIB, 9000 Ghent, Belgium; Department of Biomolecular Medicine, Ghent University, 9000 Ghent, Belgium; CSC – IT Center for Science, 02101 Espoo, Finland; ELIXIR Finland, 02101 Espoo, Finland; Turku Bioscience Centre, University of Turku and Åbo Akademi University, 20520 Turku, Finland; Institute of Biomedicine, University of Turku, 20520 Turku, Finland

This volume of *Bioinformatics* includes the proceedings papers of the 23rd European Conference on Computational Biology (ECCB2024) to be held in Turku, Finland, from 16 September to 20 September 2024, under the theme Data and Algorithms for Health and Science. More information on the ECCB2024 conference is available at the conference website https://eccb2024.fi/.

ECCB is one of the main international conferences in the field of computational biology and bioinformatics together with the Intelligent Systems for Molecular Biology (ISMB) and the Research in Computational Molecular Biology (RECOMB). It is held jointly with the ISMB conference in odd-numbered years and independently in even-numbered years. ECCB attracts scientists and industry professionals from diverse disciplines, including mathematics, statistics, computer science, biology, and medicine.

Rapid technological advancements enable life scientists to gain increasingly detailed insights in complex biological systems. These improvements in measurement technologies, however, introduce new challenges for data interpretation and necessitate the development of advanced computational techniques to manage the complexity and volume of the data. Consequently, the field of computational biology is rapidly evolving with new algorithms, software, and databases. The ECCB2024 conference showcases cutting-edge developments in systems biology, artificial intelligence, single-cell and spatial technologies, data integration, and more, addressing the growing demand for sophisticated algorithms to enhance the analysis of large-scale biological and biomedical datasets. This is highlighted by the most frequent keywords accompanying all the accepted submissions in ECCB2024 (tutorials/workshops, proceedings, highlight talks, posters), with the most popular keywords including ‘machine learning’, ‘deep learning’, ‘single-cell rnaseq’, and ‘multiomics’ (Fig. 1).

**Figure 1.**
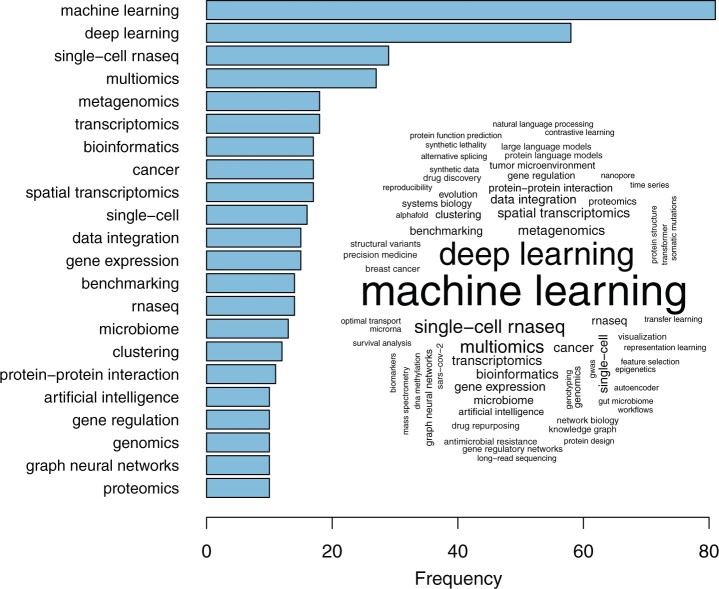
Most frequent keywords among all accepted submissions in ECCB2024. The barplot shows the frequencies of top keywords appearing in at least ten submissions, while the word cloud illustrates the relative frequencies of all keywords appearing in at least five submissions.

The ECCB2024 edition features five keynote lectures by distinguished speakers: Sarah Teichmann (Cambridge Stem Cell Institute, University of Cambridge, UK), Peer Bork (EMBL—European Molecular Biology Laboratory, Germany), Ileana Cristea (Princeton University, US), Jussi Taipale (University of Cambridge, UK), and Fabian Theis (Helmholtz Munich Computational Health Center, Germany). In addition, ECCB2024 hosts a scientific debate on data sharing and privacy protection by Melissa Haendel (University of North Carolina, US) and Yves Moreau (KU Leuven, Belgium).

The ECCB2024 conference covers a wide range of topics, focusing on methodological advancements in computational biology as well as innovative application of computational techniques to life sciences and medicine. To provide a cohesive overview of recent scientific progress, the conference presentations are organized under six broad themes: (i) Genomes, (ii) Proteins, (iii) Systems biology and multiomics, (iv) Single-cell omics, (v) Microbiomes and planetary health, and (vi) Digital health. The proceedings talks present new scientific contributions, while the highlight talks showcase already published cutting-edge science in computational biology and related further developments. Poster presentations provide an opportunity for the participants to discuss their recent work with other researchers in the field. Workshops and tutorials on specialized topics prior to the main conference program are platforms to share practical experiences and learn new skills.

In addition to scientific presentation tracks, ECCB2024 has a separate ELIXIR track, overseen by ELIXIR, focusing on advancements in infrastructure and services within ELIXIR nodes in support of the expert groups known as ‘ELIXIR Communities’. ELIXIR is a distributed pan-European life science infrastructure for biological data that coordinates, integrates, and sustains bioinformatics resources across its member states. This enables academic and industry users to access data, tools, standards, computing, and training services for life science research. ELIXIR has selected ECCB as a primary dissemination platform, serving as a co-organizing sponsor. Apart from Community activities the track will also introduce three scientific areas as outlined in the 2024–2028 ELIXIR Scientific Programme (https://elixir-europe.org), enabling scientists to access and analyse life science data across ‘Cellular and Molecular Research’, ‘Biodiversity, food security & pathogens’, and ‘Human data & translational research’.

ECCB2024 received an impressive number of 200 submissions of full manuscripts for the proceedings call, highlighting the growing interest and engagement within the community. These submissions were organized under the six conference themes. Each submission was subjected to a peer-review process, with at least two reviews per manuscript, managed by the members of the ECCB2024 Programme Committee. The Programme Committee was chaired by Laura Elo (University of Turku, Finland) and each conference theme had an area chair (Table 1). The Proceedings Review Committee had over 100 reviewers.

**Table 1.** Thematic areas of ECCB2024 proceedings talks.^a^

Theme	Area chair	Submissions	Accepted	Accept rate (%)
Genomes	Matti Nykter, Tampere University, Finland	59	5	8
Proteins	Lukas Käll, KTH Royal Institute of Technology, Sweden	37	5	14
Systems biology and multiomics	Lars Juhl Jensen, University of Copenhagen, Denmark	33	4	12
Single-cell omics	Merja Heinäniemi, University of Eastern Finland, Finland	27	4	15
Microbiomes and planetary health	Tim Van Den Bossche, Ghent University, Belgium	18	2	11
Digital health	Sampsa Hautaniemi, University of Helsinki, Finland	26	4	15

a The table lists the area chairs for each theme, the number of reviewed papers, the number of accepted papers, and the acceptance rate for each theme.

The review process focused on the impact, reproducibility, and scientific quality of the submitted research, as well as its relevance, interest, and value for the ECCB2024 audience. Upon completion of the review, the Program Committee chairs selected 24 papers to be included in the ECCB2024 proceedings, with an acceptance rate of 12% across the different areas (Table 1). All proceedings papers are published open access in this Proceedings issue of September 2024 of *Bioinformatics*.

The ECCB2024 call for Highlight Talks invited presentations of studies recently published in scientific journals since 1 March 2023, or accepted for publication. The existence of further developments related to the published paper was also positively considered. The call received a total of 212 proposals, which were ranked according to their relevance and impact on computational biology, as well as their suitability to be presented to the large and diverse audience. Ultimately, the Program Committee selected 23% of the submissions for presentation as a Highlight talk at the conference.

ECCB2024 hosts two poster sessions where researchers can introduce and discuss their work under the conference themes. In total, nearly 700 posters were accepted to be presented. The poster submissions were reviewed by the Posters Committee: Sini Junttila, Asta Laiho, Tomi Suomi, and Sampsa Hautaniemi (late posters). In addition, the ELIXIR track selected posters to be presented at ECCB2024.

Overall, the ECCB2024 tracks attracted participation of presenting authors from 48 countries. The countries with the highest number of presenting authors were Germany, Finland, the USA, the United Kingdom, and Spain, jointly contributing to half of the overall number (Fig. 2). Following these were Turkey, France, and China, each contributing 4%–5% of the presenting authors.

**Figure 2.**
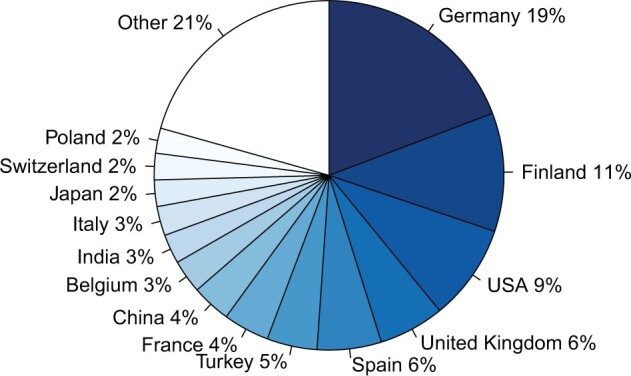
Proportion of countries where the presenting author of each accepted submission had their primary affiliation. Countries with the proportion below 2% were grouped under ‘Other’.

Exhibitor booths are open throughout the conference, including ELIXIR, ISCB, Oxford University Press, Royal Society Publishing and eLife Sciences Publications Ltd, as well as the two organizing institutions: University of Turku and CSC—IT Center for Science. The booths showcase the latest scientific literature in computational biology and bioinformatics, data stewardship, as well as new developments in hardware, software, and technology. In addition, the Visit Turku Archipelago (tourist office) is also represented.

Before the main ECCB2024 conference, a satellite meeting, seven workshops, and nine tutorials are organized. The satellite meeting is organized by the Student Council of the International Society for Computational Biology (ISCB) by and for early-stage researchers. This 8th European Student Council Symposium (ESCS) continues the successful collaboration between ESCS and ECCB, building the future of research in computational biology. The 16 workshops/tutorials preceding the ECCB2024 main conference were selected out of a total of 24 proposals by the workshops and tutorials chair Bengt Persson (Uppsala University, Sweden), each running for half a day. The workshops foster discussions and exchange of ideas on a range of specialized or emerging topics in computational biology and provide opportunities to share practical experiences. The tutorials provide participants with lectures and hands-on training to enable learning about new areas of computational biology or important established topics.

Following the tradition of previous ECCB conferences, the ISCB and ECCB sponsored a number of travel fellowships for students and postdoctoral fellows. These fellowships were primarily awarded to members presenting talks and those from low- or middle-income countries, facilitating their participation in the conference. A total of 68 applications were received from scientists across 26 countries. After a careful review, the ECCB2024 Organizing Committee in collaboration with the ISCB and ECCB awarded 10 and 18 fellowships, respectively.

The ECCB2024 Code of Conduct is designed to ensure a safe and respectful environment for all attendees, outlining clear standards of behaviour to foster an inclusive conference atmosphere. In addition, the code provides specific procedures for attendees to follow if they feel these standards have been violated. This ensures that all participants have access to support and resources to address any concerns, reinforcing the commitment of ECCB2024 to uphold the highest ethical and professional standards during the conference.

The ECCB2024 Organizing Committee is strongly committed to promote gender equity throughout the conference planning and execution. In particular, an important effort was made to ensure that the review panels included a balanced composition of female and male professionals across the world. In addition, three out of the seven distinguished keynote speakers are women. The conference program also features a collaborative workshop with the Bioinfo4Women initiative, discussing sex and gender bias in artificial intelligence.

We would like to thank everyone who has contributed to the success of ECCB2024, ensuring it meets high standards of excellence. Special recognition is due to the Program Committee, including theme area chairs and reviewers, whose critical and dedicated efforts have been fundamental to the conference organization in selecting excellent tutorials, workshops, manuscripts, talks, and posters. We are equally thankful to the ECCB steering committee for their invaluable support and advice, especially ECCB2022 organizers for providing detailed information about the organization of the previous ECCB conference. The collaboration with the ISCB has been crucial in offering travel fellowships and promoting ECCB2024 globally, for which we are deeply grateful. Our gratitude also goes to all our financial sponsors, including our co-organizing sponsor ELIXIR. We also acknowledge the Oxford University Press production team for their work on the ECCB2024 Proceedings issue.

Many individuals have played significant roles in the local organization, often exceeding their responsibilities, and we are immensely thankful for their dedication. Lastly, the conference would not be what it is without the diverse participants from around the world, whose scientific contributions, presentations, and discussions enrich ECCB2024. Thank you all for being there and for allowing us to enjoy science at ECCB2024 in Turku!

## Data Availability

All data are incorporated into the article. Additional information is available at the conference website https://eccb2024.fi.

